# Fruit host-dependent fungal communities in the microbiome of wild Queensland fruit fly larvae

**DOI:** 10.1038/s41598-020-73649-1

**Published:** 2020-10-06

**Authors:** Rajib Majumder, Brodie Sutcliffe, Phillip W. Taylor, Toni A. Chapman

**Affiliations:** 1 Applied BioSciences, Macquarie University, North Ryde, NSW 2109 Australia; 2 Department of Environmental Sciences, Macquarie University, North Ryde, NSW 2109 Australia; 3 Biosecurity and Food Safety, NSW Department of Primary Industries, Elizabeth Macarthur Agricultural Institute (EMAI), Menangle, NSW 2567 Australia

**Keywords:** Ecology, Microbiology

## Abstract

*Bactrocera tryoni* (Froggatt), the Queensland fruit fly (Qfly), is a highly polyphagous tephritid fly that is widespread in Eastern Australia. Qfly physiology is closely linked with its fungal associates, with particular relationship between Qfly nutrition and yeast or yeast-like fungi. Despite animal-associated fungi typically occurring in multi-species communities, Qfly studies have predominately involved the culture and characterisation of single fungal isolates. Further, only two studies have investigated the fungal communities associated with Qfly, and both have used culture-dependant techniques that overlook non-culturable fungi and hence under-represent, and provide a biased interpretation of, the overall fungal community. In order to explore a potentially hidden fungal diversity and complexity within the Qfly mycobiome, we used culture-independent, high-throughput Illumina sequencing techniques to comprehensively, and holistically characterized the fungal community of Qfly larvae and overcome the culture bias. We collected larvae from a range of fruit hosts along the east coast of Australia, and all had a mycobiome dominated by ascomycetes. The most abundant fungal taxa belonged to the genera *Pichia* (43%), *Candida* (20%), *Hanseniaspora* (10%), *Zygosaccharomyces* (11%) and *Penicillium* (7%). We also characterized the fungal communities of fruit hosts, and found a strong degree of overlap between larvae and fruit host communities, suggesting that these communities are intimately inter-connected. Our data suggests that larval fungal communities are acquired from surrounding fruit flesh. It is likely that the physiological benefits of Qfly exposure to fungal communities is primarily due to consumption of these fungi, not through syntrophy/symbiosis between fungi and insect ‘host’.

## Introduction

The gut microbiome plays a vital role in the metabolic regulation, food digestion and immune systems of animals^1,2^. In insects, the gut microbiome is known to contribute to the extraction of nutrients from consumed food^3^, can help to detoxify harmful compounds, and imparts protection from pathogens^4–6^. The microbes associated with insects span all three domains of life: Bacteria, Archaea and Eukaryota. The eukaryotic microbes tend to be fungal, and are predominately yeasts^7^. These yeasts play an important role in insect development and fitness, by providing nitrogen compounds and degrading high molecular weight molecules^3,8^. For example, the intracellular fungi *Symbiotaphrina* spp. has a symbiotic relationship with host beetles, assisting in the digestion of food by producing enzymes such as lipase, *α*‐ and *β*‐glucosidase, phosphatase and trypsin, and detoxifying a variety of plant toxins (e.g. 2-furaldehyde)^9,10^. Moreover, fungal spores and yeasts provide a good source of macronutrients and micronutrients to tephritid fruit flies^11^, and yeast supplements are routinely provided to laboratory-reared larvae and adult flies as a food source^12–16^. In *Drosophila suzukii* and *D. melanogaster*, yeasts affect sexual maturation, oviposition rates and larval development^17,18^. Several species of yeast also enhance survival rates, and shorten the developmental period in *D. melanogaster*^19,20^. In nature, some insects even farm fungi. For example, Brazilian stingless bees *Scaptotrigona depilis* and leaf cutter ants (two Genera, *Acromyrmex* and *Atta*) have domesticated specific strains of fungi, cultivating them as a food source^21,22^.

While the impact of yeast in insect nutrition has been relatively well studied, this is not their only role. For example, fungi may produce pheromones inside insects, which can affect communication and mating performance^23^. On the other hand, fungi are also responsible for insect disease, with more than 700 species of fungi identified as entomopathogenic^24^. For example, *Metarhizium anisopliae* fungus is pathogenic to *Drosophila*^25^. *Metarhizium anisopliae* (Metschnikoff) Sorokin and *Beauveria bassiana* (Balsamo) Vuillemin (Deuteromycotina: Hyphomycetes) both are pathogenic to adults and pupae of the Mediterranean fruit fly *Ceratitis capitata* (Wiedemann)^26–29^. It is clear from this body of research that insect-associated fungal communities have active roles in the ecology of insects, including in tephritid fruit flies. Despite this, much remains to be learned about the insect mycobiome, and the individual fungi that comprise it.

The Queensland fruit fly, *Bactrocera tryoni* (Froggatt) ('Qfly') is the most economically damaging fruit fly species in Australia^30,31^. Qfly is highly polyphagous and causes substantial economic damage to the production and trade of commercial fruits and vegetables^30–33^. In Qfly biology, the microbiome of the Qfly and its functional significance is still remains poorly understood. Several recent studies have addressed this knowledge gap, investigating the bacterial species compromising the Qfly microbiome^34–39^. Bacterial community structure of Qfly larvae is dominated by vertical transfer of the microbiome during egg laying from adult female to larvae, with comparatively minor contributions from the host fruit (diet) microbiome^34,40^. Qfly development and fitness traits were also observed using an artificial diet supplemented with microbes^41,42^. Bacterial isolates, *Enterobacter* sp. and *Asaia* sp. were supplemented with artificial larval diet shortened the development time of the Qfly larvae^42^. Furthermore, use of *Leuconostoc* sp. in artificial larval diet significantly reduced the mean time from egg hatch to adult emergence of the Qfly^42^. In addition, significant variation in bacterial communities and their abundance has been found across developmental stages of the Qfly^43,44^. In contrast with this literature focusing on bacteria, just two studies have attempted to identify the mycobiome of the Qfly, with both using traditional culture-dependent methods to isolate fungal strains^45,46^. These studies confirm the presence of *Aureobasidium, Candida, Cryptococcus, Hanseniaspora, Pichia,* and *Starmerella* fungal species in Qfly^45^. However, given the well-documented biases of culture-based microbial surveys, it is likely that a number of fungal associates remain unknown. Culture-independent molecular techniques have identified a high diversity of fungi associated with adult olive fruit fly, *B. oleae* (Gmelin)^8^, and this approach is expected to reveal a similarly diverse fungal micorbiome in the Qfly.

The molecular technique for surveying fungal communities typically involves targeting the nuclear ribosomal internal transcribed spacer (ITS) region of the RNA operon, using fungal-specific primers^47^. This region is composed of two ITS sections (ITS1 and ITS2) flanking the 5.8S region. Sequencing the ITS amplicons, and comparing them to curated databases, allows identification of which fungi are present within a given community. Using high-throughput Illumina sequencing, millions of sequence reads can be achieved at one time, although a limitation of this technique is that sequence reads are ≤ 300 bases long. Thus, surveys using high-throughput Illumina sequencing typically target either the ITS1, or ITS2 sections^48^. This technique has been used to comprehensively survey the mycobiome of insects^49–51^, along with other environments^47,52^. In the present study, we investigated the fungal community of Qfly larvae and their host fruits, using high throughput NGS analysis of the ITS1 region.

## Results

### Identification of larvae

Analysis of the mitochondrial cytochrome c oxidase subunit I (*COI*) gene confirmed that all 36 larvae collected from the different fruit type/origins were Qfly. Furthermore, all of the approximately 600 adult flies developed from the collected fruits were identified morphologically as Qfly. No other species were identified from the collected samples. Additionally, the surface sterilization process of the larvae was found to be effective as there was no microbial growth detected in different growth medias after 24–48 h incubation.

### Fungal taxa identified in Qfly larvae

**Figure 1:**
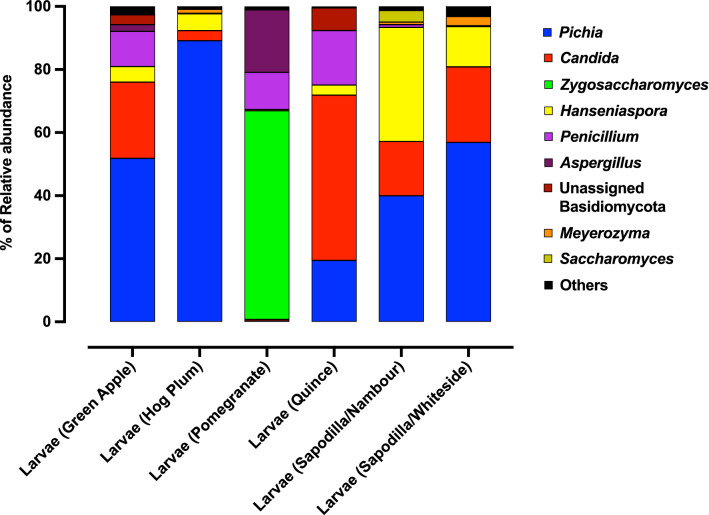
Relative abundance of fungal taxa of Qfly larvae (genus level). The percentage of relative abundance of one or less are included in “Others”. The mycobiome of larvae from each type of fruit is plotted. (6 replicates of the larvae from each types of fruit were used except pomegranate (4 replicates of the larvae from pomegranate were used).

We sequenced the mycobiome of the 36 Qfly larvae, of which 34 were retained after quality control and rarefaction at 10,000 reads per sample. Using a cluster threshold of 97% sequence similarity, 134 fungal OTUs were obtained after rarefaction (Supplementary Data S1). None of these OTUs were in all larval mycobiome samples. Of the 134 fungal OTUs detected, 102 were assigned to the Ascomycota phylum, and had an average total relative abundance of 98% (± 1% SE) in larval fungal communities. The remaining 32 OTUs belonged to the Basidiomycota and these represented an average relative abundance of just 2% (± 1% SE). Across these two phyla, OTUs spanned a total of 36 families and 37 genera, however, almost half of the sequence reads were assigned to a single genus, *Pichia,* from the Pichiaceae (45.4% ± 6.6% SE). Indeed, the *Pichia* genus was comprised of 20 OTUs, ~ 15% of the OTUs detected in larvae. Other particularly abundant families included Saccharomycetales_Incertae sedis (21.4% ± 5.6 SE), Saccharomycetaceae (8.5% ± 4.4% SE), Trichocomaceae (9.4% ± 4.2 SE) and Saccharomycodaceae (11.1% ± 3.2% SE). The fungal genera with the highest relative abundances were predominately yeasts, including *Pichia* (45.4% ± 6.6% SE), as well as *Candida* (21.4% ± 5.6% SE)*, Zygosaccharomyces* (7.8% ± 4.4% SE) and *Hanseniaspora* (11.1% ± 3.2% SE) (Fig. 1). Other abundant fungal genera were *Penicillium* (8.8% ± 2.9% SE) and *Aspergillus* (2.7% ± 2.0% SE). The remaining genera had average relative abundances of < 2%. Of these the genera detected, only three were found to be present in all larval mycobiomes: *Pichia*, *Candida* and *Hanseniaspora*.

### The fungal communities differed between Qfly larvae collected from different fruit sources

**Figure 2:**
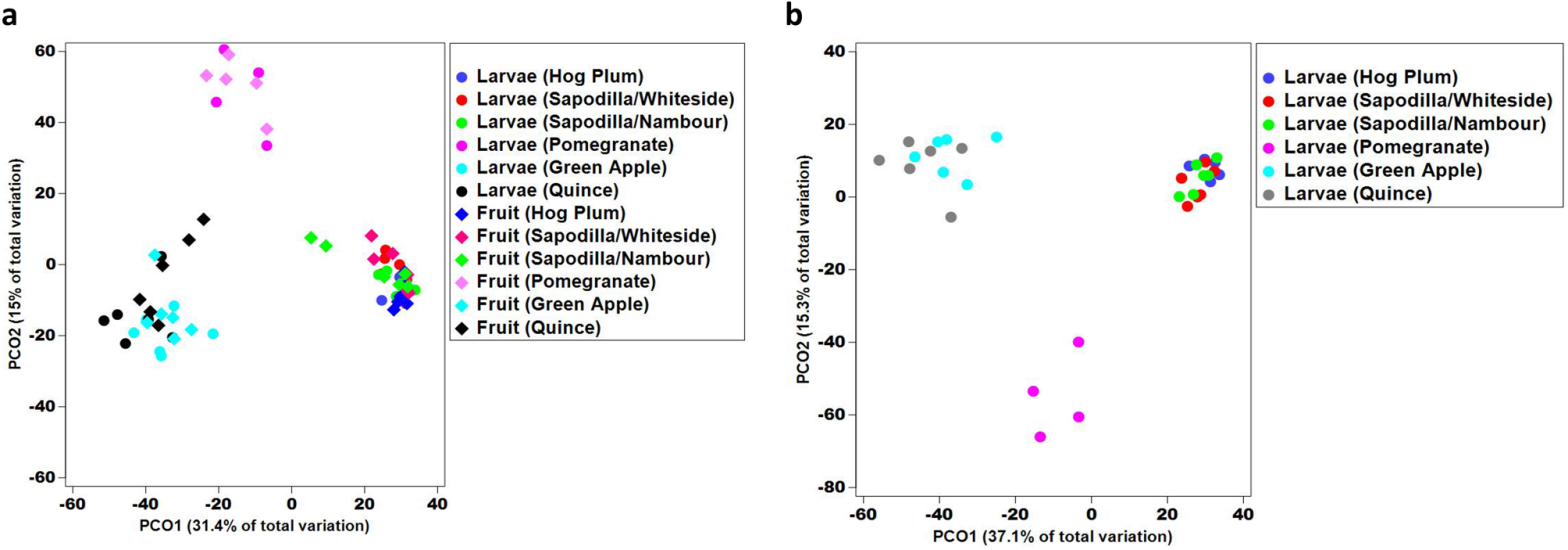
Principal co-ordinate analysis (**a**) the fungi communities of Qfly larvae from five type of fruit sources*.* (**b**) Fungal population between larvae and fruit*.* ITS high throughput NGS was performed for fungal identification. Different colour point indicates the larvae from different fruit respectively.

Fungal community ordinations of larval samples demonstrated three distinct clusters (Fig. 2a,b). Those from sapodilla larvae (two locations) and hog plum clustered closely together and were clearly separated on the first axis from those of the green apple and quince larvae. This first axis explained 37.1% of the beta diversity variation. On the second axis, which explained 15.3% of the variation, the pomegranate larvae communities were separated from all other groups. Pair-wise statistical comparisons largely supported this clustering. However, fungal communities in larvae from hog plum and the sapodilla (from two places) were found to be significantly different (Table 1), despite being clustered together in the ordination (Fig. 2a,b).

**Table 1:**
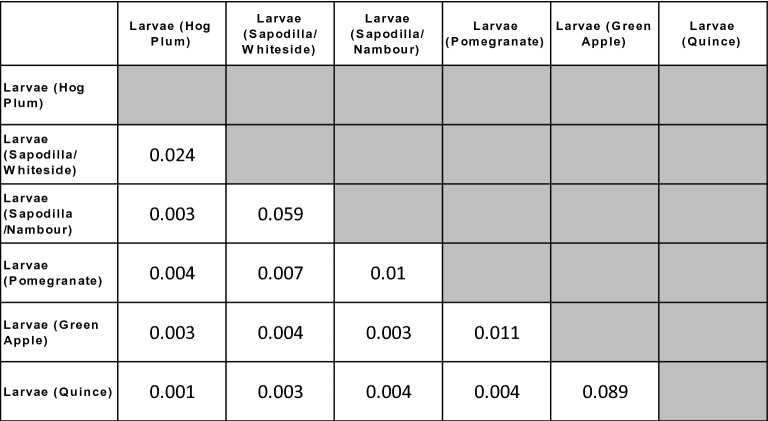
PERMANOVA test (*p* values) from Pair-wise comparisons of fungal communities between larvae from different fruits.

When interrogating the relative abundances of specific fungal genera across the larval dataset, there were clear relationships between fruit host and larval fungal associates. For larvae from hog plum, fungal communities were dominated by the genus *Pichia,* which represented an average relative abundance of 89.1% (± 4.4% SE, Fig. 1). As demonstrated by a low standard error, this high relative abundance was consistent for all hog plum larval replicates (six). *Pichia* also had a high average relative abundance in larvae collected from sapodilla in Whiteside (56.9% ± 13.0% SE) and Nambour (40.0% ± 15.3% SE), as well as green apple (51.8% ± 13.4% SE). Despite having an average relative abundance of ≥ 40% in larvae from these fruit sources, however, replicate values were sporadic for each group. For example, in larvae from green apple, relative abundances of this genera ranged from 2 to 87%. In pomegranate, *Pichia* had a consistently low relative abundance, with 1.1% in just one replicate, and all others having < 0.05% relative abundance.

Larvae from quince had a particularly high relative abundance of *Candida*, with an average of 52.4% (± 18.2% SE, Fig. 1). The genus, however, was of low relative abundance in larvae from hog plum (3.2% ± 1.7% SE) and pomegranate (0.5% ± 0.5% SE), while it was of medium relative abundance sapodilla Whiteside (24.0% ± 12.4% SE), sapodilla Nambour (17.2% ± 11.1% SE) and green apple (24.2% ± 15.2% SE). Only larvae from quince and green apple had > 1% average relative abundance of unassigned Basidiomycota (quince: 7.1% ± 4.8% SE, green apple: 3.1% ± 2.3% SE).

Larvae from pomegranate, had a uniquely high average relative abundance of *Zygosaccharomyces* (66.2% ± 22.0% SE) with no other larval group exhibiting an average abundance of > 0.05% for this genus (Fig. 1). In contrast, larvae collected from pomegranate had a very low average relative abundance of *Hanseniaspora* (0.41% ± 0.4% SE). Further, *Aspergillus* had an elevated average relative abundance of 19.8% (± 15.4% SE) in larvae from pomegranate, however, this was largely driven by a single larva in which the relative abundance of this genus was 66%. For larvae collected from sapodilla fruits, *Hanseniaspora* had an elevated relative abundance in sapodilla larvae from Nambour (36.2% ± 9.6% SE) and sapodilla larvae from Whiteside (12.7% ± 9.3% SE) compared to the other fruits (Fig. 1). Following this, the next highest average relative abundance was in hog plum, but was almost half that recorded for sapodilla (Whiteside) larvae (5.3% ± 3.7% SE).

### The fungal communities in Qfly larvae mirror that observed in fruit sources

**Figure 3:**
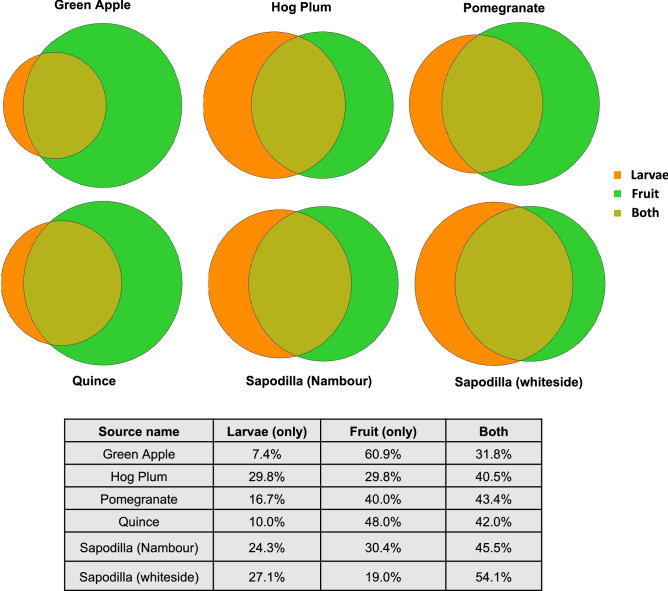
Venn diagram of the percentage of the fungi present in the larvae only, fruits only and common in both collected from five different types of fruit in the wild.

**Figure 4:**
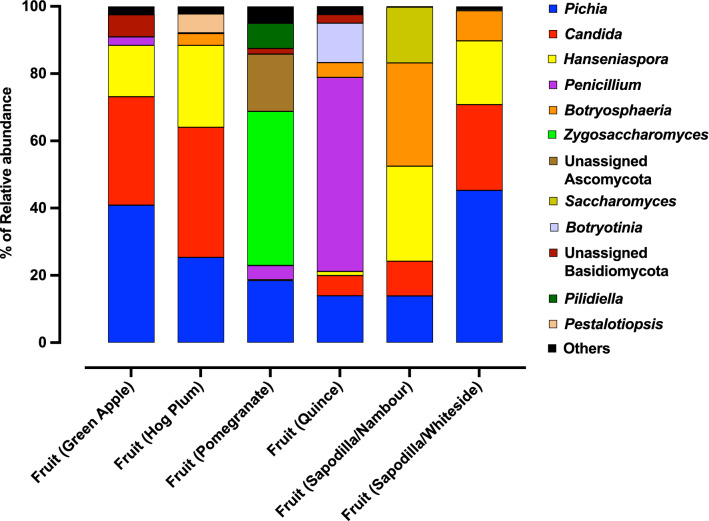
Relative abundance of fungal taxa of different types of infested fruit samples (genus level). The percentage of relative abundance of one or less are included in “Others”. The mycobiome of fruit samples from each type of fruit is plotted. (6 replicates of the fruit samples from each types of fruit were used except pomegranate (5 replicates of the pomegranate fruit samples were used).

A total of 35 samples of fruit flesh out of 36 had sufficient sequencing depth to be included in the present study (minimum of 10,000 reads) (Supplementary Data S1). A total of 191 fungal OTUs were detected from these fruit samples. Fungal community ordinations of both larval and fruit samples demonstrated the tight clustering of communities from larvae with their fruit hosts (Fig. 2a). Pair-wise statistical comparisons largely supported this finding, with only fungal communities of hog plum larvae significantly differing from those of the fruit host itself (Table 2). This overall trend is supported by the large amount of overlap in fungal communities observed when comparing larval fungal communities with their associated fruit host (Fig. 3), as well as the average relative abundances of genera (Figs. 1, 4).

**Table 2:**
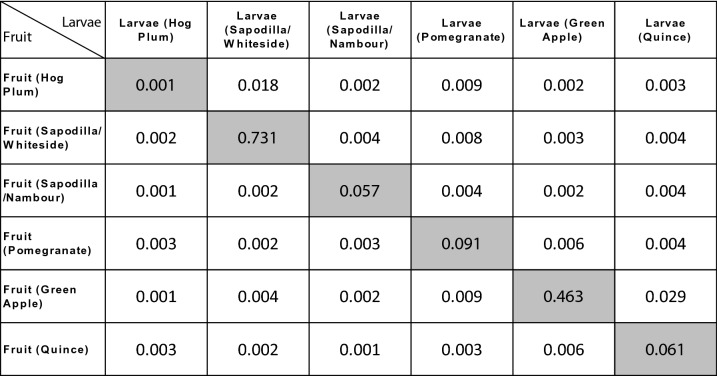
PERMANOVA test (*p* values) from Pair-wise comparisons of fungal communities between larvae and host fruits.

When comparing hog plum larval communities with fruit communities, some subtle differences can be seen in the relative abundance of specific genera. For example, hog plum fruits had higher average relative abundances for *Hanseniaspora* (24.3% ± 9.3% SE) and *Candida* (38.7% ± 8.6% SE) compared to larvae (5.3% ± 3.7% SE and 3.2% ± 1.7% SE, respectively). Conversely, there was a clear enrichment of *Pichia* in the larval fungal communities, with an average relative abundance of 89.1% (± 4.4% SE), compared with the fruit (25.3% ± 3.5% SE, Figs. 1, 4). None of these individual trends, however, were found to be statistically significant. Of the genera detected in fruit tissues, only two were found to be present in all fruit mycobiomes: *Pichia* and *Candida* (Fig. 4).

## Discussion

The present study is the first comprehensive analysis of the mycobiome of Qfly larvae using high throughput Illumina sequencing. In addition to this, it is the first study to couple the mycobiome analysis of Qfly larvae with that of the fruit host, providing insights into the likely role of host fruit mycobiome in shaping the Qfly larval mycobiome. In this study, after collection of infested fruit from nature, we waited until the larvae had developed to 3^rd^ instar before sampling. The Qfly larvae were not starved prior to sampling. The larvae were directly removed from the infested fruit and processed the sample for the mitochondrial cytochrome c oxidase subunit I (*COI*) gene identification and high-throughput Illumina sequencing. Transient and resident (including pathogenic) microbiota are both components of the overall microbiome. It is useful to consider which microbes are transient and which are resident. However, it takes several days to clear the gut of starved larvae, and many are unable to tolerate this treatment. In addition, starvation not only clears the gut but also changes the gut environment in ways that can influence which microbes persist. In this study, we have compared the prevalence of each microbe type in the larval samples and in the fruit tissues. Those that are identifiably more prevalent in the larva can be highlighted as likely resident.

Our results in part support the findings of previous culture-based studies^45,46^, in that we observed that *Pichia* were diverse and ubiquitous in the mycobiome of Qfly larvae. Previously, this genus has also been observed in *Drosophila*, beetles, and lepidopteran larvae^50,53–55^. Additionally, the *Pichia* are known to be common in sugar rich environments such as ripe fruits^50^. Thus, the presence of *Pichia* in the larval mycobiome is well supported by the literature. However, the present study finds that the relative abundance of *Pichia* in the Qfly larval mycobiome is dependent on host fruit source. For example, *Pichia* dominated the mycobiome of larvae from hog plum, representing ~ 90% of the community, but was rare in larvae from pomegranate (≤ 1% relative abundance).

In addition to supporting the findings of previous research, the use of culture-independent methods in the present study has substantially extended knowledge of the Qfly mycobiomes and provided the first insights to the relationship between the host and the Qfly mycobiomes. For example, we identified fungal genera that were previously unknown members of the Qfly larval mycobiome including *Zygosaccharomyces, Penicillium* and *Aspergillus*. While *Penicillium* and *Aspergillus* were sporadically detected, the *Zygosaccharomyces* were a signature component of the pomegranate larval mycobiome. This yeast genus has previously been detected in the mycobiome of the bumblebee^10,53^, however, it was not ubiquitous across all larvae in the current study and thus, is unlikely to represent a member of the insect core mycobiome. Instead, the presence in larvae from pomegranate appears to be diet-specific in Qfly. Certainly, diet is an essential factor influencing the gut microbial community of other insects; e.g., cotton bollworm *Helicoverpa armigera*^56^, ground dwelling beetles (Coleoptera)^57^, gypsy moth, *Lymantria dispar* L.^58^, and *Drosophila*^59^.

The high similarity between mycobiome in larvae and their respective host fruit source suggests that these fungal communities are closely interconnected. This is in direct contrast to previous work with bacteria^40^, which demonstrated that larval microbiome communities were quite distinct from those of the host fruit. This previous work suggested a distinct bacterial “environmental niche” in the host larvae, and proposed that these bacteria are acquired through vertical transfer from adult Qfly females. If Qfly females vertically transfer fungal associates to their larvae, our results suggest that the fungi are subsequently dispersed throughout the fruit flesh, either independent of the larvae or assisted by larval burrowing behavior. Insect-assisted fungal dispersal has been reported previously in the literature^53,60,61^ and such a strategy could enhance the nutrition for larvae. This enhancement may be two-fold, for example, given that fruit fly larvae are known to feed on yeast in fruit^19^, this strategy would provide yeast for larval consumption. In this study, infested fruits were mostly over-ripe and collected from under trees. As we worked with wild samples, it is not possible to know the precise time of infestation and the starting time of fungal growth in infested fruit. However, because the fruit were all in a state of infestation and decomposition at time of collection, we can be confident that the mycobiome had ample opportunity to develop. Further, we collected all of the larvae samples at the 3rd (final) instar, thereby allowing further time for the mycobiome to develop and to interact between the larvae and the fruit. Additionally, yeast is known to release nutritional components from the fruit substrate by producing extracellular enzymes^62^ and this may make nutrients further accessible to the larvae. Such a relationship would be beneficial to both Qfly and fungi, representing a symbiosis despite the fact individual fungi are consumed by Qfly larvae.

An alternative explanation for the interconnected larvae and fruit mycobiomes is that Qfly females do not vertically transfer fungi to larvae. Instead, the fungi identified in our study may have colonized the fruit independently, and under such circumstances, the larvae would acquire the fungi through horizontal transfer from the fruit. This would mean that the larvae are wholly dependent on the dispersal and environmental filters shaping fungal colonization of a given fruit for their own survival and development, a potentially risky life-strategy. At this time, not enough is known about the dependence of Qfly larvae on their mycobiome in general, or of particular types of fungi, to assess the extent of such risk or the extent to which fungi are functionally equivalent. Despite this, the poor overlap between mycobiomes from different fruit host sources supports such an explanation. Indeed, none of the OTUs detected were observed in all larval samples and of the three genera which were (*Pichia*, *Candida* and *Hanseniaspora*), large variations in relative abundance occurred across the dataset.

Interestingly, of the six fruit host sources, five had statistically similar mycobiomes in hosts and larvae, but one did not. This exception was in the hog plum, where a substantive enrichment of *Pichia* was observed for the larvae compared with the fruit. One explanation for this trend is that the larvae might have a distinct dietary preference for particular fungi^10^. This has been observed in other insects, including olive fruit fly *Bactrocera oleae* (Gmelin)^8^, and ground dwelling beetles (Coleoptera)^57^. It might be possible that there are differential survival rates for different fungi in the larvae, with *Pichia* having a longer survival time in larvae than other community members. Given that *Pichia* was detected in all fruits, however, these explanations do not explain why a similar enrichment was not observed in the larvae from the other fruits. Perhaps larvae in hog plum require greater nutritional supplementation from *Pichia* compared with larvae from the other fruits.

## Conclusion

We investigated the extent to which Qfly larvae and hosts share a common microbiome, and the extent to which the mycobiome of larvae and hosts varies across host fruit. Qfly larvae are found to harbor a diverse range of yeasts, most of which are also found in the host fruit. This relationship persisted despite massive variation in the mycobiome of different host fruit types and associated larvae, and demonstrates close relationship between the two communities. Our findings provide valuable insights for understanding the ecology of Qfly, in particular this species’ ability to infest a vast diversity of fruit types. Our findings also have implications for insect health during laboratory and factory-scale rearing.

## Methods

### Collection of Qfly larvae

Qfly larvae were obtained from infested fruits collected from various geographic locations within Australia, including the states of New South Wales (NSW), Victoria (VIC) and Queensland (QLD) (Table 3). Most infested fruits were over-ripe and were collected from under trees. The fruit types included: Hog Plum *Spondias mombin* L., Sapodilla *Manilkara zapota* (L.) P. Royen (from two different localities, Nambour and Whiteside, QLD), Pomegranate *Punica granatum* L., Green Apple *Malus pumila* Borkh., and Quince *Cydonia oblonga* Mill. (Table 1). The infested fruits were stored on wire racks in plastic bins (60 L, 447 × 236 × 663 mm, Award, Bunnings Warehouse, Greenacre, NSW, Australia) that contained a 1 cm deep layer of fine vermiculite (Grade 1, Sage Horticultural, Hallam, VIC, Australia) in a controlled environment laboratory (25 ± 0.20 °C, 65 ± 3% RH and 11:1:11:1 light:dusk:dark:dawn photoperiod). Samples of different fruit types and origins were kept separate to prevent cross-contamination. We collected *B. tryoni* larvae (3rd instar) from each of the six replicate fruits from each of the five fruit types (36 larval samples in total). Additionally, six replicate samples of fruit tissue (fruit flesh) (1–2 mg mass) were collected from the same fruit used to collect larvae (36 fruit samples in total).

**Table 3:** Fruit types and origin for Qfly larvae collection.

Geographic location of collection	Fruit source and number of fruits collected	Collection date
Maroochy Research station, Nambour, QLD GPS: Lat 26° 38′ 34.92″, Long 152° 56′ 22.99″	Hog Plum 26 pieces	1/02/17
Daboro Road, Whiteside, QLD, 4503 GPS: Lat 27° 14′ 29.31″, Long 152° 55′ 8.49″	Sapodilla 52 pieces	1/02/17
Maroochy Research station, Nambour, QLD GPS: Lat 26° 38′ 34.92″, Long 152° 56′ 22.99″	Sapodilla 68 pieces	1/02/17
Commealla, NSW GPS: Lat 34° 5′ 50.97″, Long 142° 3′ 7.21″	Pomegranate 37 pieces	5/05/17
St. Germains, Between Tatura and Echuca in Victoria GPS: Lat 36° 10′ 48.86″, Long 145° 8′ 50.74″	Green Apple 41 pieces	05/05/17
Downer road between Tatura and Toolamba in Victoria GPS: Lat 26° 38′ 34.92″, Long 152° 56′ 22.99″	Quince 52 pieces	05/05/17

A total of six replicate larvae, and fruit flesh samples were collected from each fruit origin.

### Sample preparation

Larvae were surface sterilized with a solution of 0.5% (v/v) Tween 80 (Sigma-Aldrich, St. Louis, MO, USA, Cat. No. 9005656), 0.5% (v/v), Bleach (sodium hypochlorite) (Sigma-Aldrich, St. Louis, MO, USA, Cat. No.7681529) and 80% (v/v) Ethanol (Sigma-Aldrich, St. Louis, MO, USA, Cat. No. 65175) for 30 s, and were rinsed 3 times in 1 M sterile phosphate-buffered saline (1 × PBS) for 30 s after collection^40,44,45^. To check the performance of the surface sterilisations, 100 µL of the 2nd and 3rd washes of PBS were collected and plated onto the five growth medium plates and incubated at 32 °C and 35 °C for 24 to 48 h respectively^40,44^. The five types of microbial growth medium were: de Man, Rogosa and Sharpe Agar, Tryptone Soya Agar, Macconkey Agar, Potato Dextrose Agar and yeast dextrose Agar medium (Sigma-Aldrich, St. Louis, MO, USA). After sterilization, sterile pestles (Thermo Fisher scientific, Waltham, MA, USA) were used to crush the whole larvae, which were then stored in Brain Heart Infusion (BHI) broth (Oxoid Ltd, Basingstoke, UK, Lot # 1656503) with 20% Glycerol (Sigma Aldrich, St. Louis, MO, USA, Lot # SHBG2711V) solution at – 80 °C. The samples were split into two separate cryovial tubes (Simport Scientific, Saint-Mathieu-de-Beloeil, QC, Canada) for mitochondrial cytochrome c oxidase subunit I (*COI*) gene identification and high-throughput Illumina sequencing^40,44^. Fruit flesh from individual fruit was also preserved and stored under the same conditions. All procedures were completed in a sterile environment (Biological Air Clean Bench, safe 2020 1.2, Thermo Scientific, Dreieich, Germany).

### Larval identification

Microscopic examination of larval morphology was carried out prior to DNA extraction. All the defining morphological traits were examined to identify the larvae^40,63^. Further, larval identification was confirmed by sequencing the mitochondrial cytochrome c oxidase subunit I (*COI*) gene of all samples. DNA was extracted from crushed whole larval samples using Isolate II genomic DNA kit (Bioline, Taunton, MA, USA. Cat. no. BIO-52066) following the manufacturer’s protocol. Standard LCO1490/HCO2198 primers were used to amplify a 700 bases segment of the *COI* gene^40,44,64^ in triplicate PCRs. The final reaction volume was 15 µL and was comprised of 7.5 μL of MyTaq HS PCR master mix (Bioline, Taunton, MA, USA. Cat No. BIO-25045), 0.60 µL of forward (LCO1490F) and 0.60 µL of reverse primer (HCO2198R), 1.5 μL of DNA extract and 4.8 µL of nuclease-free water. All PCR amplifications were performed using an Eppendorf thermocycler (Mastercycler, epgradient S, Eppendorf, Germany) using the following thermal cycling conditions: an initial denaturing step at 95 °C for 2 min, followed by 35 cycles of 94 °C for 30 s, 50 °C for 30 s and 72 °C for 90 s, and a final extension step of 72 °C for 5 min^40,44^. Amplicons were visualised using electrophoresis on a 1% agarose gel (110v-45 min)^40,44^, before being sent to the Australian Genomic Research Facility (AGRF) (University of Adelaide, Plant Genomics Centre, Hartley Grove, URRBRAE, SA 5064, AU) for Sanger sequencing. Sequence data were analysed by Geneious R10.2.3 using NCBI database^65^ to confirm Qfly larvae identification. Additional confirmation was also gained through examination of adult morphology after the emergence of adult flies (approximately 600) from the larvae remaining in the infested fruits using a stereomicroscope (Leica MZ6 stereo-microscope, Leica, Wetzlar, Germany)^40,44,66^.

### Mycobiome profiling

DNeasy PowerLyzer PowerSoil Kit-100 (Qiagen, Hilden, Germany, Cat. no. 12855-100) was used to complete the DNA extraction process of the crushed whole larvae samples for high-throughput Illumina sequencing following the manufacturer’s protocol. Invitrogen Qubit dsDNA High Sensitivity (HS) Assay Kit (Life Technologies, Eugene, OR, USA) were used to quantify the DNA extracts^40,44^. The AGRF performed the PCR amplification and sequencing procedures. In brief, the ITS1 region of rRNA operon was amplified using the fungal-specific forward primer ITS1F (5′-CTTGGTCATTTAGAGGAAGTAA-3′) and the reverse primer ITS2 (5′-GCTGCGTTCTTCATCGATGC-3′)^44,48,52,67,68^. Reactions contained 1X AmpliTaq Gold 360 mastermix (Life Technologies, Eugene, OR, USA), 0.20 µM of forward and reverse primers and 25 µL of DNA. PCR cycling conditions included denaturation at 95 °C for 7 min, followed by 35 cycles of denaturation at 94 °C for 45 s, annealing at 50 °C for 60 s and extension at 72 °C for 60 s, with a final extension at 72 °C for 7 min. A second PCR was used to adhere sequencing adaptors and indexes to the amplicons. Primerstar max DNA Polymerase was used to generate a second PCR amplicon from Takara Bio inc., Shiga, Japan (Cat. No. #R045Q). The resulting amplicons were measured by fluorimetry (Invitrogen Picogreen, Thermo Fisher Scientific, NSW, Australia) and normalized^69^. Equimolar amounts of each sample were pooled and quantified by qPCR prior to sequencing (Kapa qPCR Library Quantification kit, Roche, Basel, Switzerland)^40^. The resulting amplicon library was sequenced on the Illumina MiSeq platform (San Diego, CA, USA) with 2 × 300 base pairs paired-end chemistry^70^.

### Sequence data processing

The Greenfield Hybrid Amplicon Pipeline (GHAP) (version: GHAP. v1.CSIRO) was used to process amplicon sequences^52,71^. The Greenfield Hybrid Amplicon Pipeline (GHAP) is a publically available amplicon clustering and classification pipeline (https://doi.org/10.4225/08/59f98560eba25) built around tools from USEARCH^72^ and Ribosomal Database Project (RDP)^73^. It was combined with locally written tools for demultiplexing and generating OTU (operational taxonomic units) tables. This hybrid pipeline took files of reads and produced tables of classified OTUs and their associated read counts across all samples. The amplicon reads were demultiplexed, the read pairs merged, de-replicated and removed low quality reads and chimeric sequences. The merged reads were trimmed and clustered at 97% similarity to generate OTUs^52^.

Representative sequences from each OTU were classified both by finding their closest match in the Warcup reference set of ITS sequences, and by using the RDP Naïve Bayesian Classifier and the Warcup training set^74^. The use of two independent classification techniques can improve confidence in the taxonomic assignments. This process highlights those cases where a simple ‘best match’ might give a misleading result. Each OTU sequence was also classified with the RDP Classifier and compared with the UNITE^74,75^ training set to increase confidence in the classifications.

The pipeline mapped the merged reads back onto the classified OTU sequences to obtain accurate read counts for each OTU/sample pairing and generated OTU tables complete with taxonomic classifications and species assignments. The OTU tables summarised overall taxonomic levels and combined the counts for identified taxa across all OTUs. The pipeline finally classified all the merged reads using the RDP Classifier, regardless of whether they were assigned to an OTU. This last step was carried out to provide confidence in the clustering and OTU formation steps by providing an independent view of the community structure. Each OTU was tested using the following criteria to ensure that OTU sequences were actually fungal. RDP taxonomic assignments using UNITE or Warcup included an assignment to a fungal order with > 60% confidence. Alternatively, Blastn returned a similarity of > 70% using the Geneious software (Geneious 10.2.3, Biomatters Ltd.) to match with NCBI Insights fungal reference set.

### Statistical analysis

The OTU table containing the number of read counts for each OTU detected for each sample was imported into Primer-E v7 for analysis^44,52,76^. In brief, all statistical testing was performed on fixed factors associated with fruit host (hog plum, sapodilla [from two different localities], pomegranate, green apple and quince) from which 6 replicates were collected. The DIVERSE function was used to generate univariate biodiversity metrics, including total species, species richness, and Shannon’s biodiversity indices. Statistical differences between these metrics were assessed in JMP Statistical Software Version 10.0.0 (SAS Institute, Cary, NC, USA) using one-way analysis of variance (ANOVA) and Tukey–Kramer’s HSD post hoc analysis^40,44^.

The OTU table was first log transformed using Primer-E V7 to observe the taxonomic compositional changes for fungal communities. A Bray–Curtis similarity matrix was derived from these transformed data and a permutational analysis of variance (PERMANOVA) pair wise comparison was conducted to compare all community samples. A p value of < 0.05 was considered statistically significant. Further, ordination plots of these communities were visualised using principal coordinates analysis (PCoA) in Primer-E. Fungal Taxonomic plots for larvae and host fruit were modelled in Prism 8 (version 8.0.1(145), GraphPad software, Inc)^40,44^.

## Supplementary information

Supplementary Legend.

Supplementary Data.

## Data Availability

OTU sequences were deposited and currently available in the NCBI GenBank under Bio-project PRJNA647994.
