# Supplementary material for: Fruit host-dependent fungal communities in the microbiome of wild Queensland fruit fly larvae
Source: Sci Rep. 2020 Oct 6;10:16550. doi: 10.1038/s41598-020-73649-1 (PMC7538879; doi:10.1038/s41598-020-73649-1)
Supplement: Supplementary file 1 — Supplementary Legend. [file 41598_2020_73649_MOESM1_ESM.docx]

**Supplementary information**

**Fruit Host-dependent Fungal Communities in the Microbiome of Wild Queensland Fruit Fly Larvae**

*Rajib Majumder^1,3*^, Brodie Sutcliffe^2^, Phillip W. Taylor^1^ & Toni A. Chapman^1,3^*

*^1^* *Applied BioSciences, Macquarie University, North Ryde, NSW 2109, Australia*

*^2^Department of Environmental Sciences, Macquarie University, North Ryde, NSW 2109, Australia
^3^Biosecurity and Food Safety, NSW Department of Primary Industries, Elizabeth Macarthur Agricultural Institute (EMAI), Menangle, NSW 2567, Australia*

**Running head:** Mycobiome analysis of Queensland fruit fly larvae

To whom correspondence should be addressed:

Rajib Majumder* (Main Correspondence)

Applied BioSciences, Macquarie University, North Ryde, NSW 2109, Australia

Email: rajib.majumder@mq.edu.au

**SUPPLEMENTARY MATERIAL**

Supplementary Data 1: OTU Table & Mapping file
